# Ion and Molecule Transport in Membrane Systems 2.0

**DOI:** 10.3390/ijms22073533

**Published:** 2021-03-29

**Authors:** Victor Nikonenko, Natalia Pismenskaya

**Affiliations:** Membrane Institute, Kuban State University, Krasnodar 350040, Russia; n_pismen@mail.ru

In this book, the papers published in the second issue, “Ion and Molecule Transport in Membrane Systems 2.0”, are recollected. As we mentioned in the editorial to the first issue collection, biological membranes constitute an integral part of any living organism. The significance of artificial membranes increases from year to year. These membranes are the heart of environment-friendly technologies of water treatment, including the production of drinking water and waste water treatment with fractionation and removal of harmful and valuable components; with the possibility to reuse the latter. In addition, many other membrane-based technologies are built in medicine (such as artificial organs, drug delivery), in eco-friendly energy production, in microfluidics, and other areas. In this special issue, the papers aimed at physicochemical and chemico-physical aspects of ion and molecule transport in the systems involving biological or artificial membranes are collected. The scope of the issue includes the same items as the first issue.

As in the first issue, both biological and artificial membranes and corresponding membrane systems continue to be the focus of attention in this issue.

The transport mechanisms in biomembrane systems are studied in [1,2,3,4]. The complicated transfer of keratinocyte toward the wound skin was studied by S. Hwang [1]. This study revealed that the directionality of Ca^2+^-exerted stimulation can play an important role in facilitating migration of keratinocyte by involving anion exchanger AE2. Decoding this mechanism can greatly help in wound healing. The effect of campylobacter concisus (which is a pathogenic epsilonproteobacterium, causing enteritis and diarrhea) on the intestinal transport function was investigated by P.K. Nattramilarasu et al. [2]. The mechanism of the impact of campylobacter concisus causing Na^+^ malabsorption and diarrhea was studied using a cell model and mouse colon. A physico-chemical approach to describe the mechanisms of the principle of minimum information loss by living organism was developed by B. Frieden and R. Gatenby [3]. Heritable information in the genome causes long-term changes in the cellular macromolecules. In contrast, the reaction of a cell to the received information is rapid and continuous. The authors assume that these two information dynamics are linked. Mathematical analysis of information dynamics is based on the flow of ions through membrane channels and along wire-like cytoskeleton macromolecules, when assuming that minimization of signal loss is a mechanism to overcome energy constraints. S.Y. Jung et al. [4] made a review of the relationship between the properties of aquaporins (which are water-specific membrane channel proteins) and rhinologic disorders, such as nasal mucosa and olfactory mucosa, rhinosinusitis, and others.

There are publications aimed at the preparation and study of novel artificial membranes [5,6,7,8,9,10]. M. Mofasserul Alam et al. [5] prepared and studied a novel anion-exchange membrane designed to separate bisulfite anion from mixt solutions. The thermal-induced phase separation method for grafting a quaternized moiety was applied. The membrane showed excellent substance stability in an alkali medium and high HSO_3−_ separation performance. V. Sarapulova et al. [6] examined the interplay between the ion exchange capacity, water content, and concentration dependences of such transport properties as conductivity, diffusion permeability, and counterion permselectivity for a series of anion-exchange membranes, the production of which was recently launched by Hefei Chemjoy Polymer Materials Co. Ltd., China. Experimental results were analyzed using the microheterogeneous model, opening up the structure–properties relationships for these membranes. The study of S. Nanthapong [7] is devoted to membrane separators applied in zinc–air batteries, which represent an alternative to lithium–ion batteries. A new composite membrane based on polyvinyl alcohol and containing an embedded ion-conducting mobile composition as a filler was prepared and studied. The obtained results suggest that the new composite membrane is promising for use as a separator in some types of zinc–air batteries. The effect of electrical heterogeneity of an ion-exchange membrane surface on mass transfer and water splitting during electrodialysis of a 0.02 M NaCl solution was studied by S. Zyryanova et al. [8]. A series of membrane samples with 13 different patterns (representing fluoropolymer spots of different shapes and sizes) on their surface was tested. It was found that the maximum rate of mass transfer and minimum rate of water splitting (undesired effect) were achieved when the spot size was close to that of the diffusion layer thickness (about 250 μm under the experimental conditions), and the fraction of the screened surface was about 20%. It was found that the main cause of improving membrane behavior after the surface modification is electroconvection. The effect of the nature of hydrophobic material on the operation parameters of membrane contactors used to remove ammoniacal nitrogen (N-NH_4_) molecules from wastewater was studied by B. Brennan et al. [9]. It was found that polypropylene membranes are incompatible with the condensate waste, while the polytetrafluoroethylene membranes showed high performance in removing up to 64% of NH_3_ molecules from the condensate waste. The developed process can be used for the production of fertilizers from wastewater. The ability of a series of cellulose-triacetate-based polymer inclusion membranes (PIMs) to transport Ag (I) was studied by A. Nowik-Zajac et al. [10]. The membranes differed by different concentrations of a calixpyrrole ester derivative as the membrane carrier. The Ag(I) transport was assessed as a function of the concentration of metal ions, the pH of the source aqueous phase, and stripping agents, as well as the presence of other cations (such as Cu (II), Pb (II), and others) in the source solution.

Structure–properties relationships in membrane systems were studied in [11,12]. M. Izquierdo-Gil [11] studied the relationship between NaCl electrolyte permeability and water content for three sulfonated cation-exchange membranes largely used in the practice of electro-membrane processes: MK-40, Nafion N324, and Nafion 117 membranes. A close to linear correlation was established: the higher the water content, the greater the electrolyte permeability. The Nafion N324 was found to be the least permeable to electrolytes. I. Stenina et al. [12] delivered a review focusing on the ion-exchange membranes’ structure–properties relationships. The following correlation was analyzed: the higher the flux density of the target component through the membrane, the lower the selectivity of the process. Two aspects of this correlation were examined: the trade-off between membrane permeability and permselectivity, and the impact of the concentration polarization. Recent approaches aimed at membrane improvement, such as crosslinking, nanoparticle doping, surface modification, and special synthetic methods, are considered.

Some papers have been devoted to the investigation of the physicochemical aspects of different membrane processes. An interesting contribution to our knowledge about the application of electrodialysis with bipolar membrane (EDBM) to the separation of whey proteins was made by C. Aspirault et al. [13]. EDBM is a green process that simultaneously provides acidification and demineralization of a solution without adding any chemical compounds. It was found that preheating of the source solution to 60 °C allowed the purity of isolated proteins (such as β-lactoglobulin) and their recovery yield to be increased. N. Pismenskaya et al. [14] examined the mechanisms of adsorption of anthocyanins (which is a strong natural antioxidant) by cation- and anion-exchange resins from aqueous solutions at different pH values. It was shown that the kinetic and equilibrium of adsorption can be understood when taking into account that the pH of the internal solution of a cation-exchange resin is two to three units lower, while that of an anion-exchange resin is two to four units higher than the pH of the external solution. Different electrostatic interactions between anthocyanins and resin functional groups occur depending on the pH of the internal solution.

There are two theoretical papers describing ion and water transport in membrane systems. An irreversible thermodynamic approach similar to that of Kedem and Katchalsky (who first derived that to describe bio-membrane transport, see the editorial to the first issue) was applied by W. Kujawski et al. [15] to describe ion and water transport in reverse electrodialysis. The latter is an electro-membrane process harvesting electricity from the salinity gradient (e.g., from mixing river and sea water across a membrane). A simplified equation system obtained in [15] describes the power density generated in the system with good accuracy. J. Schiffbauer et al. [16] studied how the presence of hydraulically permeable pores in an ion-selective membrane affects the transitions from underlimiting to overlimiting current and the occurrence of electrokinetic instabilities leading to intensification of electroconvection. The latter is convective transport of solution under the action of an applied electric field. The Nernst–Planck–Poisson–Stokes equation system was used in the solution, and the Darcy–Brinkman approach was employed in the nanoporous membrane. Note that the significance of electroconvection was established experimentally by S. Zyryanova et al. [8].
